# Machine Learning in the Differentiation of Soft Tissue Neoplasms: Comparison of Fat-Suppressed T2WI and Apparent Diffusion Coefficient (ADC) Features-Based Models

**DOI:** 10.1007/s10278-021-00513-7

**Published:** 2021-09-20

**Authors:** Peian Hu, Lei Chen, Zhengrong Zhou

**Affiliations:** 1 Department of Radiology, Children’s Hospital of Fudan University, National Children’s Medical Center, No.399, Wanyuan Road, 201102 Shanghai, China; 2 Department of Radiology, Minhang Branch, Fudan University Shanghai Cancer Center, No. 106, Ruili Road, Shanghai, 201100 China; 3 Department of Radiology, Fudan University Shanghai Cancer Center, Fudan University, No. 270, Dongan Road, Shanghai, 200032 China; 4 Department of Oncology, Shanghai Medical College, Fudan University, No. 270, Dongan Road, Shanghai, 200032 China

**Keywords:** Soft tissue neoplasms (STN), Least absolute shrinkage and selection operator (LASSO), Texture analysis (TA), Apparent diffusion coefficient (ADC), Diffusion MR weighted imaging

## Abstract

Machine learning has been widely used in the characterization of tumors recently. This article aims to explore the feasibility of the whole tumor fat-suppressed (FS) T2WI and ADC features-based least absolute shrinkage and selection operator (LASSO)-logistic predictive models in the differentiation of soft tissue neoplasms (STN). The clinical and MR findings of 160 cases with 161 histologically proven STN were reviewed, retrospectively, 75 with diffusion-weighted imaging (DWI with *b* values of 50, 400, and 800 s/mm^2^). They were divided into benign and malignant groups and further divided into training (70%) and validation (30%) cohorts. The MR FS T2WI and ADC features-based LASSO-logistic models were built and compared. The AUC of the FS T2WI features-based LASSO-logistic regression model for benign and malignant prediction was 0.65 and 0.75 for the training and validation cohorts. The model’s sensitivity, specificity, and accuracy of the validation cohort were 55%, 96%, and 76.6%. While the AUC of the ADC features-based model was 0.932 and 0.955 for the training and validation cohorts. The model’s sensitivity, specificity, and accuracy were 83.3%, 100%, and 91.7%. The performances of these models were also validated by decision curve analysis (DCA). The AUC of the whole tumor ADC features-based LASSO-logistic regression predictive model was larger than that of FS T2WI features (*p* = 0.017). The whole tumor fat-suppressed T2WI and ADC features-based LASSO-logistic predictive models both can serve as useful tools in the differentiation of STN. ADC features-based LASSO-logistic regression predictive model did better than that of FS T2WI features.

## Introduction

Soft tissue neoplasms (STN), a group of heterogeneous tumors, are derived from blood vessels, lymphatic vessels, nerves, muscles, or other connective tissue [1]. STNs are commonly seen with complicated components and classified as benign, intermediate (metastatic or recurrent occasionally), and malignant subtypes by the WHO [1]. Except for a few tumors with characteristic imaging features, a definite histological diagnosis is usually challenging on imaging. A better prognosis can be achieved for most benign and intermediate STN. Soft tissue sarcoma (STS) represents about 1% of all malignancy; it recurs and metastasizes commonly with a poor prognosis [2]. 

MR imaging is the preferred method for detecting and staging of STN [3–6]. Conventional MR assessment of STN mainly focused on the morphologic findings, such as the tumor’s size (> or ≤ 5 cm), contour (round or lobulated), margins (well- or ill-defined), heterogeneity of masses, and involvement of adjacent vital structures (bone/neurovascular bundle) [3–5, 7, 8]. Several studies were designed to explore the effectiveness of conventional MR in the differentiation of STN. The reported diagnostic accuracy ranged from 50 to 90% [3, 5, 6, 8, 9]. An overlap of the radiological features between benign and malignant tumors was frequently seen. Gadolinium (Gd)-based enhanced MR scan helped differentiate cystic from solid masses [10]. Additionally, the knowledge of prevalence and presentation of onset can serve as a supplement of morphological features in the differentiation of STN [3].

Surgical excision was the first-choice treatment for STN. Although the role of chemotherapy was controversial [11], a few subtypes of sarcomas were sensitive to chemotherapy, such as rhabdomyosarcoma (embryonal and alveolar subtypes), Ewing sarcoma family of tumors, round cell liposarcoma, desmoplastic small round cell tumor, and synovial sarcoma [11].

Diffusion magnetic resonance weighted imaging (DWI) based on the Brownian motion of water molecules can reflect the tissue microstructures [12]. The apparent diffusion coefficient (ADC) is a widely used quantitative parameter. Low ADC values mean highly cellular density and/or restricted microenvironments, while acellular regions are found with elevated ADC values [12–15]. Muscular sarcomas were reported with a broad range of ADC values [16]. Some researchers thought that ADC value was a reliable quantitative parameter in the differentiation of STN [13, 14, 17].

Texture analysis (TA) is a method to evaluate the tumor by extracting and using features that were invisible to the naked eye. Texture analysis was employed to differentiate tumors or tumors with different grades be employed to differentiate tumors or tumors with different grades [18–20] but scarcely did they focus on the application of TA based on FS T2WI and ADC mapping in the differentiation of STN. Machine learning, as the intersection of statistics and computer science, has been gradually applied in the medical field recently [21]. It mainly focused on how computers learn from big data and included many algorithmic models, such as the least absolute shrinkage and selection operator (LASSO), support vector machine (SVM), random forest, and decision tree [22–24]. LASSO was commonly used and robust. It overcame the shortcomings of multiple regression in high-dimensional data and was beneficial in feature selection [23–25]. 

We supposed that the TA of the whole tumor FS T2WI and ADC features-based LASSO-logistic regression predictive models can be used in the characterization of STN precisely. An then to assess the effectiveness of these two models in the characterization of STN, we retrospectively collected and reviewed the clinical and imaging findings of 160 patients with 161 histologically proven STN (75 of them with DWI).

## Methods

### Study Population

This retrospective study was approved by our institutional review board, and informed consent was waived. Between July 1, 2015, and December 31, 2015, the imaging features and clinical findings of patients with suspected soft tissue neoplasms were collected and reviewed retrospectively. The inclusion criteria were as follows: STN were all histologically proven (surgery or biopsy), and all the patients underwent an MR scan. The suspected STN that were not histologically proven or without MR scans were excluded. 

At last, 160 cases (161 histologically proven masses) with MR scans were collected and reviewed, and 75 of them with diffusion-weighted imaging (DWI, with *b* values 50, 400, and 800 s/mm^2^). The 38 soft tissue sarcoma (STS) cases with DWI were divided three times, into the chemosensitive and non-chemosensitive groups [11]; the small round cell and non-small round cell sarcoma groups; and the rhabdomyosarcoma and non-rhabdomyosarcoma groups.

### Demographic and Clinical Data

The demographic and clinical data were reviewed, including the age of onset, gender, main manifestations, tumor locations, and histological results. The locations were recorded as the head and neck, trunk, retroperitoneum, and extremities, respectively.

### Imaging Acquisition

**Fig. 1:**
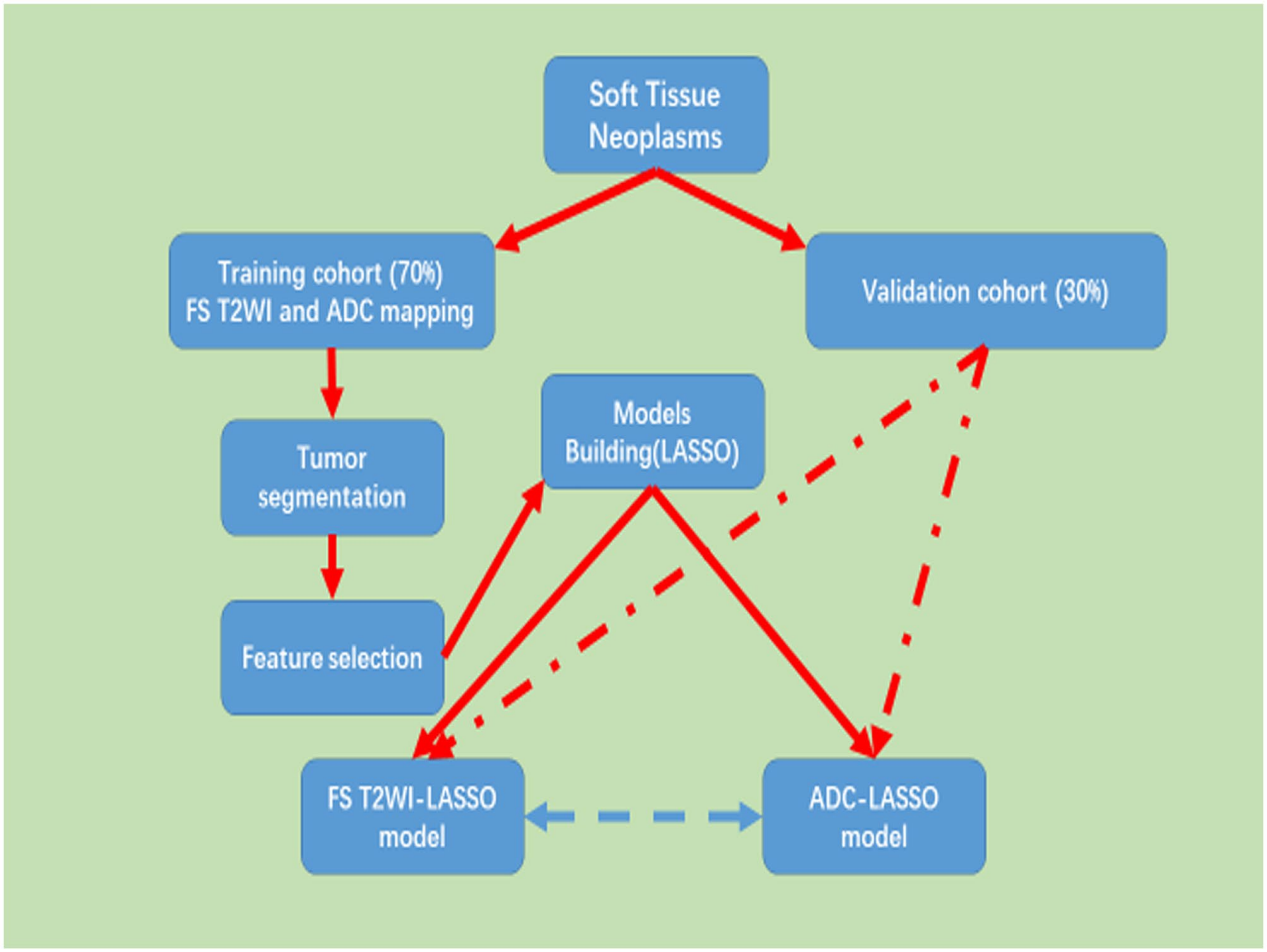
Flowchart of radiomics and machine learning

**Fig. 2:**
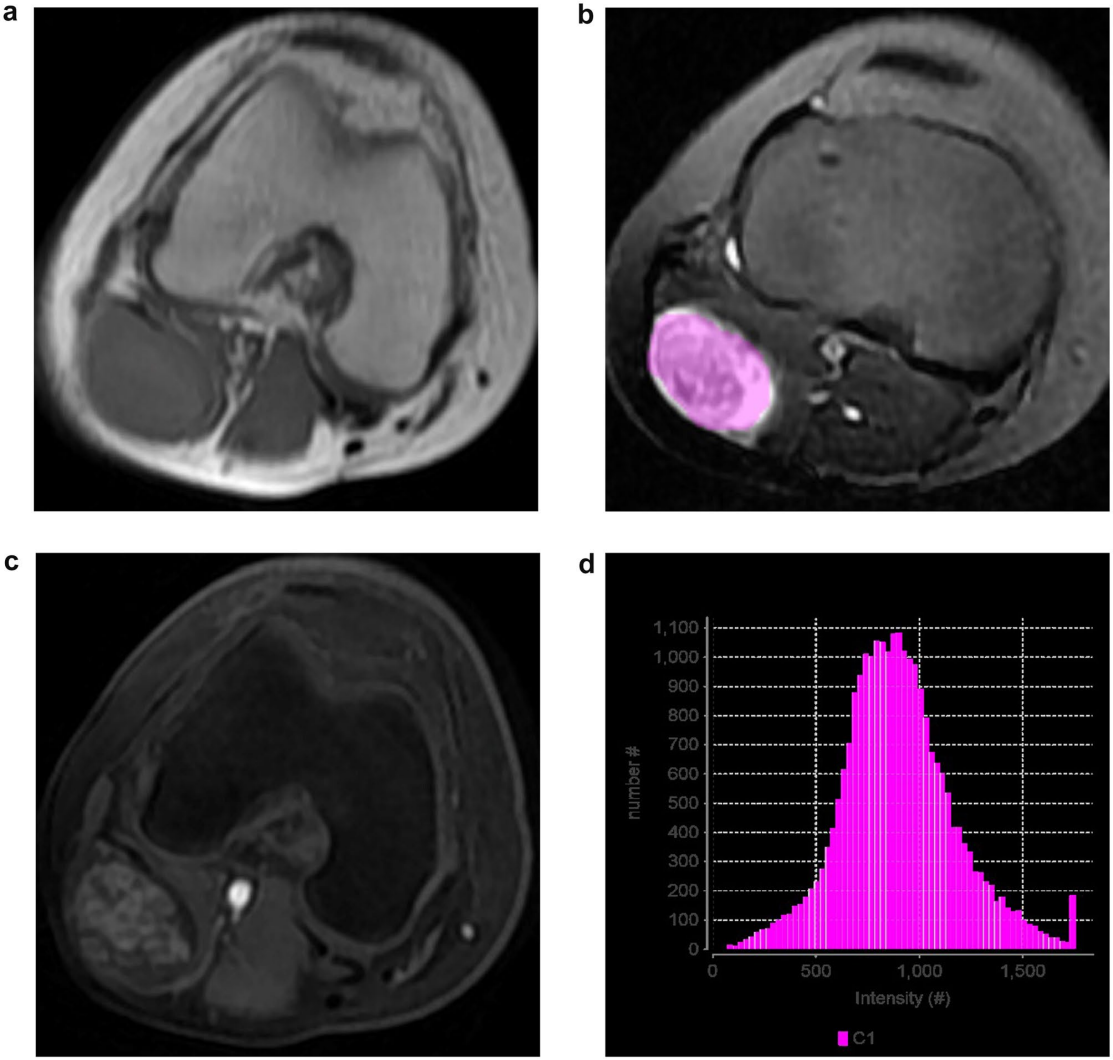
**A** 19-year-old female with a schwannoma in the right popliteal fossa. **a**–**c** showed iso-signal intensity on MR T1WI (**a**), heterogeneous hyper-signal intensity (SI) on MR FS (fat-suppressed) T2WI (**b**), and moderate heterogeneous enhancement (**c**). **d** the FS T2WI SI distribution of the whole tumor

**Fig. 3:**
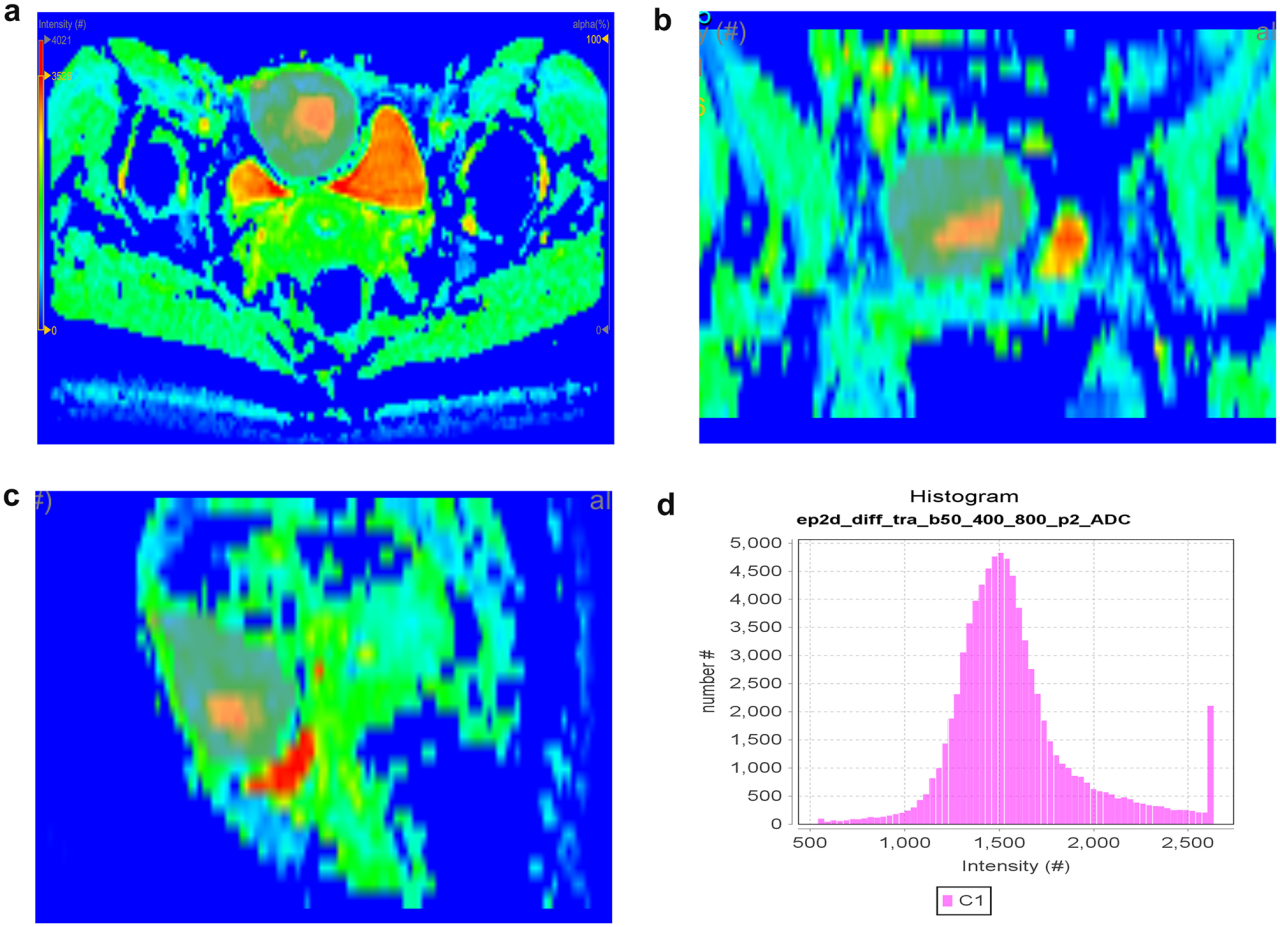
A 29-year-old female with fibrosarcoma in the anterior abdominal wall showed 3D manually selected ROI on color ADC map (**a**–**c**) and histogram distribution of whole tumor ADC values (**d**)

All the patients underwent conventional MR and/or DWI (with *b* values of 50, 400, and 800 s/mm^2^). Axial FS T2WI imaging and/or ADC mapping was used for whole tumor 3D volume segmentation and feature extraction (Figs. 1–3): 

- The scanned FS T2WI parameters: TR 3,500–4,000 ms, TE 100–110 ms, ETL 15, matrix 512 × 512, the number of excitation 2, the slice thickness 5 mm, the gap of slice 1 mm, and FOV 250–350 mm
- T1WI: axial FSE/TSE sequences, TR 410–500 ms, TE 15 ms, matrix 512 × 512, the number of excitation 2, slice thickness 5 mm, and the gap of slice 1 mm
- T2WI: coronal or sagittal TSE/FSE, TR 3,500–4,000 ms, TE 100–110 ms, the number of excitation 2, the slice thickness 5 mm, and the gap of slice 1 mm

DWI was performed before enhanced T1WI. DWI was acquired by using the single-shot echo-planar imaging pulse (SS-SE-EPI)-DWI sequence in free breathing with parallel imaging, with *b* values of 50, 400, and 800 s/mm^2^. Other scanning parameter was the same as that described above. The ADC mapping was generated using the mono-exponential decay mode.

Subsequently, all patients underwent enhanced T1-weighted imaging after the intravenous injection of 0.1 mmol/kg contrast medium (Magnevist, Bayer Schering Pharma, Berlin, Germany) at a flow rate of 2–3 ml/s.

### Tumor Segmentation and the Extraction of FS T2WI and ADC Features

LIFEx v4.00 software (https://www.lifexsoft.org/) was employed for tumor segmentation and feature extraction.

Tumor segmentation was done by a radiologist with 12 years of experience on MR interpretations of STN (Figs. 2 and 3). Conventional MR images were referred to during selection of the region of interest (ROI).

The ROIs were manually selected using LIFEx v4.00 software, to cover the whole tumor. The steps of texture feature extraction were as follows: ROI selection (3D model), spatial resampling (1 mm × 1 mm × 1 mm), intensity discretization (number of Gray-level, 64), and intensity rescaling (relative, mean ± 3SD). The ROIs were measured twice at a 1-year interval.

These features were extracted (Table 1), including first-order features (the shape (Sphericity, Compacity, Volume_ml, and Volume_vx), histogram (HISTO_Skewness, HISTO_Kurtosis, HISTO_Entropy_log10, HISTO_Entropy_log2, and HISTO_Energy) (Figs. 2b and 3a), and second-order features (GLCM (GLCM, Gray-level co-occurrence matrix) (GLCM_Homogeneity, GLCM_Energy, GLCM_Contrast, GLCM_Correlation, GLCM_Entropy_log10, GLCM_Entropy_log2, and GLCM_Dissimilarity), GLRLM (GLRLM, Gray-level run length matrix) (GLRLM_SRE, GLRLM_LRE, GLRLM_LGRE, GLRLM_HGRE, GLRLM_SRLGE, GLRLM_SRHGE, GLRLM_LRLGE, GLRLM_LRHGE, GLRLM_GLNU, GLRLM_RLNU, and GLRLM_RP), NGLDM (NGLDM, Neighboring Gray-level dependence matrix) (NGLDM_Coarseness, NGLDM_Contrast, and NGLDM_Busyness), and GLZLM (GLZLM, Gray-level zone length matrix) (GLZLM_SZE, GLZLM_LZE, GLZLM_LGZE, GLZLM_HGZE, GLZLM_SZLGE, GLZLM_SZHGE, GLZLM_LZLGE, GLZLM_LZHGE, GLZLM_GLNU, GLZLM_ZLNU, and GLZLM_ZP)).

**Table 1:** The histological type and numbers (cases) of two groups

Group	Histological type and number
Benign	Hemangioma 22, schwannoma 19, desmoid tumor 8, lymphangioma 5, benign soft tissue neoplasms 5, inflammatory myofibroblastic tumor 5, kaposiform hemangioendothelioma 3, intermediate tumor 3, lipoblastoma 2, leiomyoma 2, nodular fasciitis 2, giant cell tumor of tendon sheath 2, angiomyxoma 1, dermatofibrosarcoma protuberans 1, fibroma 1, myxoma 1, hemangiopericytoma 1, fibrous hamartoma 1
Malignant	Rhabdomyosarcoma 23, malignant fibroblast/myofibroblast origin tumor 12, leiomyosarcoma 9, malignant mesenchymal tumor with uncertain origin 7, GIST 7, MPNST 4, liposarcoma 4, malignant solitary fibrous tumor 3, pleomorphic undifferentiated sarcoma 2, PNET 1, malignant rhabdomyoid tumor 1, malignant hemangiopericytoma 1, mesenchymal chondrosarcoma 1, clear cell sarcoma 1, malignant mucinous tumor 1

### The Construction and Validation of the Predictive Model

These cases were randomly divided into training (70%) and validation (30%) cohorts. The texture features of the training cohort were used for constructing the predictive model, and the features of the validation cohort were used for validation.

The inter-observer correlation coefficient (ICC) was used to evaluate the repeatability of these features. In order to handle high-dimensional data better and select features, the LASSO algorithm was employed. LASSO-logistic regression with tenfold cross-validation and 1 standard error rule was used to reduce data dimensions, select features, and build a predictive model. The receiver operating characteristic (ROC) and DCA were used to validate the effectiveness of the model.

### Statistical Analysis

The R (version 3.6.0, https://www.r-project.org/), SPSS 20.0, and MedCalc statistical software were employed for data analysis. Kolmogorov–Smirnov test was employed for testing normal distribution. Independent student’s *t* test was employed to analyze the differences in texture features. ROC curves were generated to determine the cut-off values. The AUCs were calculated and further compared by the Delong test. The DCA was done by R software. The glmnet and pROC packages of R software were employed. Values of *p* < 0.05 were considered statistically significant.

## Results

### Demographic and Clinical Data

There were 84 masses in the benign and 77 in the malignant group (Table 1). And there were 37 benign and 38 malignant STNs with DWI.

The gender ratio (female:male) was 77:83. The ages ranged from 1 month to 82 years old, and the median age was 29.5 years old. Thirty-three were in the head and neck region, 93 arise in the trunk (7 in retroperitoneal space), and 35 arisen in the extremity (21 in the lower, 14 in the upper).

In the malignant group, metastases were found in 23.4% (18/77) cases; 13 pulmonary metastasis, 3 liver metastasis, 3 lymph nodule metastasis, and 3 intraperitoneal dissemination were found.

There were 38 cases with STS that underwent DWI; 17 chemosensitive and 21 non-chemosensitive sarcomas, 13 small and 25 non-small round cell sarcomas, and 17 rhabdomyosarcomas and 21 non-rhabdomyosarcomas were enrolled.

Most of them complained of enlarging, pain, or painless masses. The other manifestations were the Kasabach–Merritt phenomenon (KMP), proteinuria (1 case), and yellowish skin.

### The Differences of MR FS T2WI and ADC Features Between Benign and Malignant Groups

The ICC of texture features ranged from 0.81 to 0.94, showing good repeatability.

There were 14 MR FS T2WI features with significant differences between benign and malignant STN (*p* < 0.05) (Table 2). And there were 12 features between benign and malignant tumors (*p* < 0.05) with significant differences, including mean ADC, max ADC, STD value, and HISTO-skewness values (Table 2).

**Table 2:** Differences of MR FS T2WI and ADC features between benign and malignant groups

Sequences	Features (*p* < 0.05)	
MR FS T2WI	First-order	SHAPE_VolumemL, SHAPE_Volume#vx
	Second-order	GLCM_Homogeneity, GLCM_Contrast, GLCM_Correlation, GLCM_Dissimilarity, GLRLM_GLNU, GLRLM_RLNU, GLRLM_RP, NGLDM_Contrast, NGLDM_Busyness, GLZLM_SZE, GLZLM_SZHGE, GLZLM_ZP
ADC	First-order	Mean value, max value, STD value, HISTO_Skewness
	Second-order	GLCM_Energy, GLCM_Contrast, GLCM_Dissimilarity, GLRLM_LRE, GLRLM_LRHGE, NGLDM_Contrast, GLZLM_HGZE, GLZLM_ZP

The mean ADC value (1668.93 ± 406.34 μm^2^/s), max ADC value (3167.38 ± 711.68 μm^2^/s), and STD value (374.54 ± 110.21) of benign tumors were higher than those of malignant ones (1173 ± 289.65 μm^2^/s, 2746.72 ± 823.17 μm^2^/s, 293.32 ± 102.60, *p* ≤ 0.021). Compared with benign tumors (−0.16 ± 0.58), malignant tumors had a higher HISTO-skewness value (0.52 ± 0.60, *p* = 0.000).

The cut-off values of the mean ADC value, max ADC value, STD value, and HISTO-skewness value for differentiation were 1388.55 × μm^2^/s, 2568.88 × μm^2^/s, 309.02 × μm^2^/s, and 0.29, respectively. The AUC of the mean ADC value (0.868, 95% CI, 0.771–0.935) was bigger than that of max ADC value (0.653, 95% CI, 0.534–0.759) and STD value (0.717, 95% CI, 0.601–0.815) (*p* < 0.0001, *p* = 0.0026). There were no significant differences between the AUC of mean ADC value and that of HISTO-skewness (0.791, 95% CI, 0.682–0.876) (*p* = 0.1624).

The features between chemosensitive and non-chemosensitive sarcomas, between small round and non-small round cell sarcomas, and between rhabdomyosarcomas and non-rhabdomyosarcomas were not significantly different (*p* > 0.05).

### The Construction and Validation of FS T2WI and ADC Features-Based Predictive Models

LASSO algorithm with tenfold cross-validation was employed for reducing data dimensions and feature selection.

**Fig. 4:**
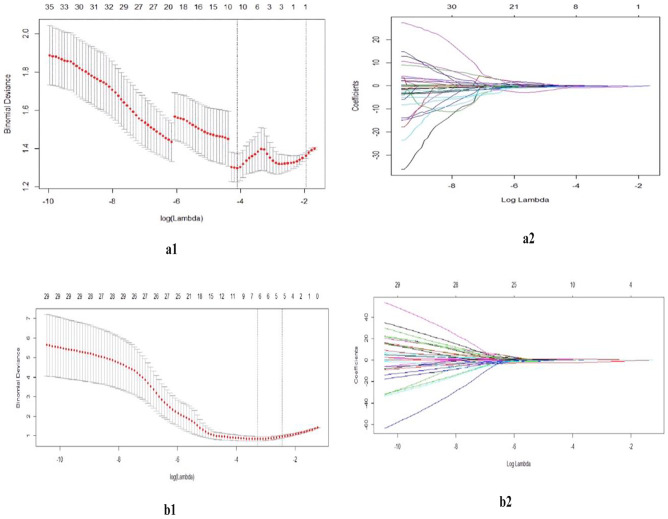
Feature selection using the LASSO-logistic algorithm using tenfold cross-validation and 1 standard error rule. The optimal tuning value (**a1**, **b1**) was selected for benign and malignant STN prediction and (**a2**, **b2**) the corresponding features

The whole tumor 3D MR FS T2WI features of the training cohort (114 cases) were used to build predictive models. The deviance of classification was minimized when the λ (lambda) was 0.134 (Fig. 4). And only one feature, GLZLM_ZP, was selected. The LASSO-logistic regression predictive model was built and the linear regression equation was *Y*_*benign/malignant*_ =  *−0.0713–0.2472* × *(GLZLM_ZP)*.

**Fig. 5:**
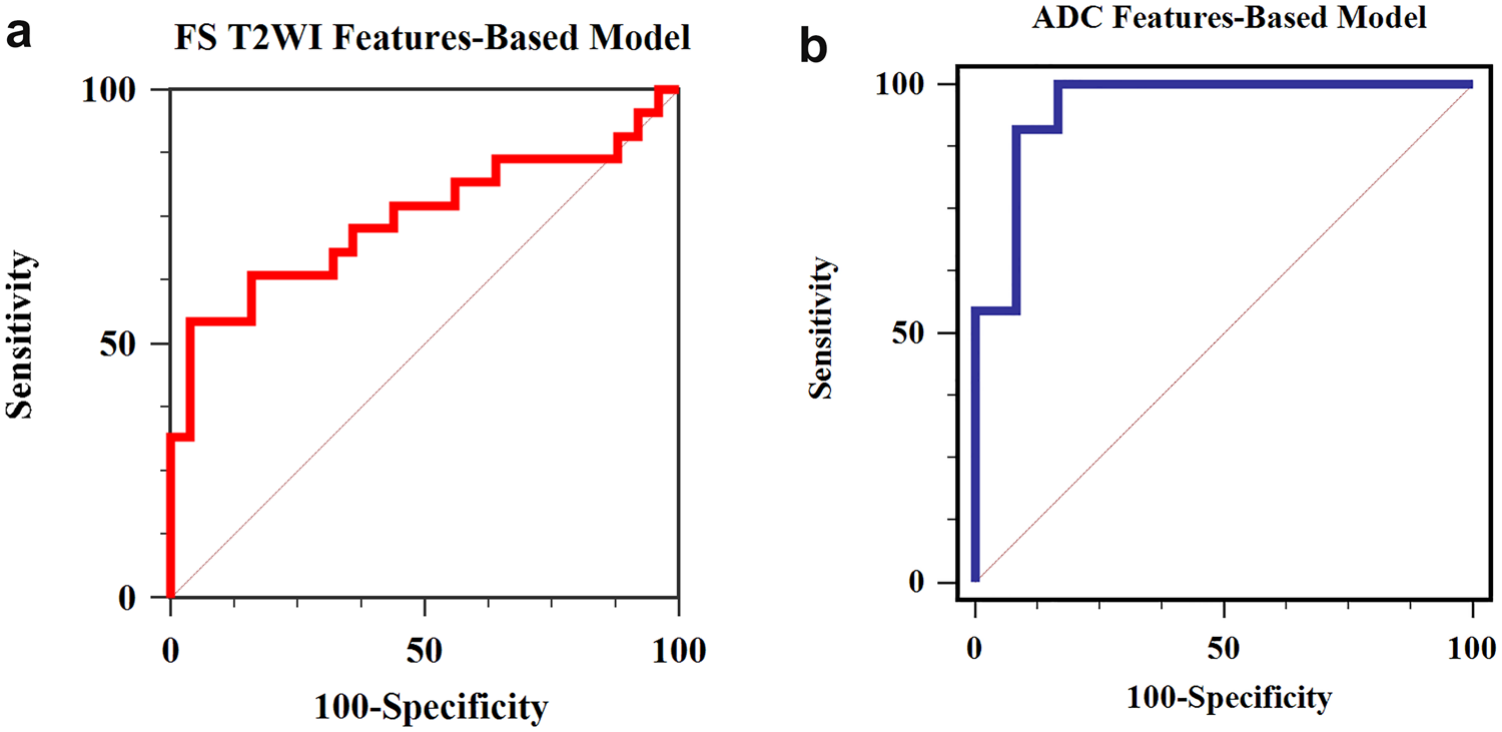
The ROC curve of benign and malignant STN differentiation in validation cohorts, the ADC features-based LASSO-logistic regression predictive model did better than that of the FS T2WI features-based model (z = 2.386, *p* = 0.017). **a** FS T2WI features-based predictive model (AUC, 0.75). **b** ADC features-based predictive model (AUC, 0.955)

The AUC of the ROC curve was 0.65 for the training cohort. The AUC of the ROC curve was 0.75 for the validation cohort (Fig. 5a), and the sensitivity, specificity, and accuracy were 55%, 96%, and 76.6%, respectively.

The deviance of classification was minimized when the lambda (λ) was 0.038 (Fig. 4). Meanwhile, six features (mean ADC value, HISTO-skewness value, GLCM_Contrast, GLCM_Energy, GLRLM_LRHGE, and GLZLM_ZP) were selected. The LASSO-logistic regression model was constructed, and the regression equation was *Y*_*benign/malignant*_ =  *−0.0615–1.9112* × *(mean ADC value)* + *0.0959* × *(HISTO_Skewness value)* + *0.0534* × *(GLCM_Energy) −0.0811* × *(GLCM_Contrast)* + *0.7319* × *(GLRLM_LRHGE) −0.151* × *(GLZLM_ZP)*.

The AUC was 0.932 for the training set. The AUC was 0.955 for the validation set (Fig. 5b), and the sensitivity, specificity, and accuracy were 83.3%, 100%, and 91.7% respectively.

**Fig. 6:**
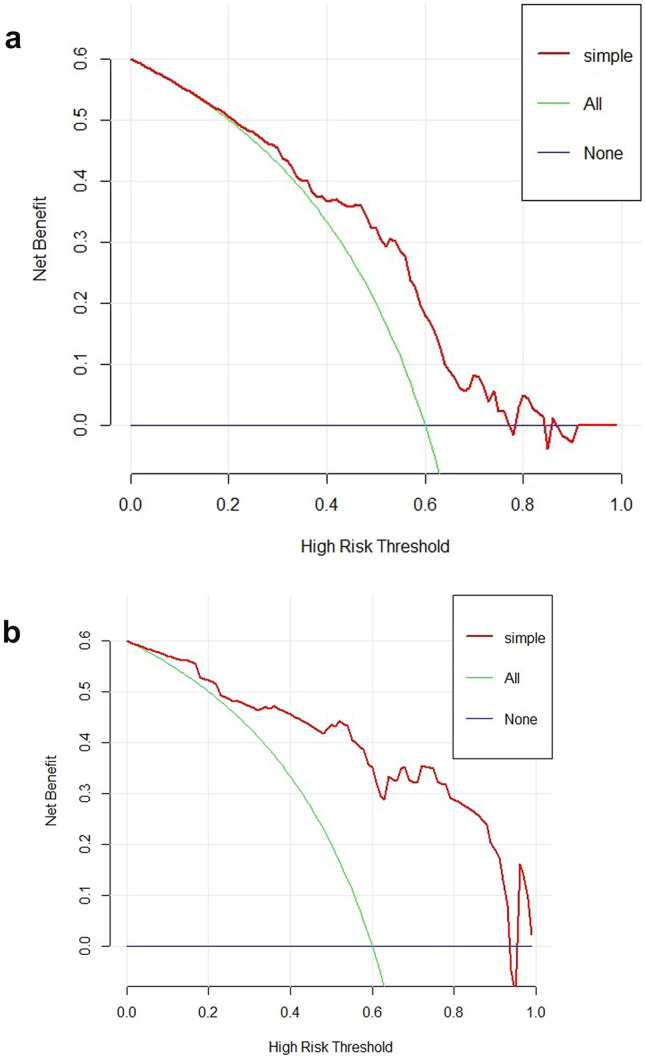
DCA showed the predictive models provided greater benefit for benign and malignant characterization. **a** FS T2WI features-based predictive model. **b** ADC features-based predictive model

The effectiveness of the predicted models was also validated by DCA (Fig. 6). DCA of FS T2WI and ADC features-based predictive models showed that these two models provided greater benefit for benign and malignant characterization.

### The Comparison of FS T2WI and ADC Features-Based Predictive Models

The ROCs of validation cohorts were used for the comparison of FS T2WI and ADC features-based predictive models. The ADC features-based LASSO-logistic regression predictive model did better than that of the FS T2WI in the differentiation of STN (*z* = 2.386, *p* = 0.017).

## Discussion

The whole tumor ADC value was not helpful in the differentiation of chemosensitive and non-chemosensitive sarcomas, small round and non-small round sarcomas, or rhabdomyosarcomas and non-rhabdomyosarcomas. The mean ADC value did better than max ADC value and STD value in the differentiation of STN. The HISTO-skewness value can be served as another useful feature in the differentiation. Machine learning of the whole tumor FS T2WI and ADC values did facilitate the differentiation of benign and malignant STN. And ADC features-based LASSO-logistic regression predictive model did better than that of FS T2WI features.

Texture analysis by extracting indiscernible radiomic features was useful in analyzing tumor heterogeneity. The utilization of images can be maximized without adding scan sequences [20]. Corino VDA et al. found that MR radiomic features can be used to distinguish intermediate soft tissue sarcomas from high-grade ones accurately [26]. The accuracy and AUC were 0.90 and 0.85 and 0.88 and 0.87 for the validation and test sets. Although we found the FS T2WI features-based model with high specificity (96%), the sensitivity was low (55%). The ADC features-based model can achieve high effectiveness. The sensitivity, specificity, and accuracy were 83.3%, 100%, and 91.7%, respectively. And ADC features-based LASSO-logistic regression predictive model did better than that of FS T2WI features.

ADC value was affected by ROI position and selected *b* values. We selected the whole tumor as ROI to avoid the selected bias. The quantitative parameter we measured showed good repeatability. Similar to literature [14], we chose three *b* values (50, 400, and 800 s/mm^2^). For *b* = 50 s/mm^2^, it was less affected by microvascular perfusion than *b* = 0 s/mm^2^, and the selection of 800 s/mm^2^ was to ensure enough signal-to-noise ratio (SNR).

DWI reflecting water molecule diffusion is useful in the detection and differentiation of tumors and facilitates the therapeutic assessment [13, 27–32]. Some benign STN resembled malignant ones on conventional MR sequences and were usually misdiagnosed [31, 33]. Most researchers thought the mean ADC and minimal ADC values help in the differentiation of STN [34, 35]. The mean ADC value of volumetric quantification had a high inter-observer agreement and reflected tumors’ heterogeneity [36]. Although Van Rijswijk CSP et al. (37) harbored different opinions, they thought that malignant ones had significantly lower true diffusion coefficients. We found the mean ADC and HISTO-skewness values were valuable in the characterization of STN and did better than minimal ADC values. And it was tested by the LASSO-logistic model. The HISTO-skewness value can be served as another useful feature in differentiation, which was not mentioned previously. Benign STN often exhibited a negatively skewed distribution due to their low cell density and large extracellular space, while the malignant ones showed a positively skewed distribution. TA of ADC mapping can acquire more quantitative or semi-quantitative features for the differentiation of STN.

Several limitations should be mentioned. Selective bias could not be avoided; these patients were relatively younger, and the rhabdomyosarcoma was the most common malignancy. The sample size of intermediate tumors was relatively small. Those tumors seldom metastasize or recur and therefore were classified as benign. The value of texture analysis in the differentiation of STN should be explored at different anatomic sites. Considering the sample size, we did not compare the efficacy of different machine learning models. Moreover, the point-to-point radiological and histological correlation couldn’t be done, due to the retrospective property.

## Conclusion

ADC features of the whole tumor couldn’t differentiate chemosensitive from non-chemosensitive sarcomas, small round from non-small round sarcomas, or rhabdomyosarcomas from non-rhabdomyosarcomas. The mean ADC and HISTO-skewness values did help in differentiating benign from malignant STN.

The ADC features-based LASSO-logistic predictive model did better than the FS T2WI features-based model in the characterization of STN.

## Data Availability

The raw data can be made available.

## Code Availability

The R, SPSS 20.0, and MedCalc statistical software were used.
